# New Jersey State Dental Society

**Published:** 1884-11

**Authors:** 


(TO BE CONTINUED.)

---

### NEW JERSEY STATE DENTAL SOCIETY.

### FOURTEENTH ANNUAL MEETING AT ASBURY PARK, JULY 16, 17 AND 18, 1884.

REPORTED FOR THE INDEPENDENT PRACTITIONER.

(Continued from page 584.)

*Dr. James G. Palmer*—Read a paper entitled "Higher Dental Attainments." (See page 621.)

*Dr. R. Finley Hunt*—I must, in the first place, congratulate the State of New Jersey, and the Dental Society of New Jersey, on the fact that they have a law now which embraces that feature which should be embodied in all laws in all the States, and that is that no person, after the date of the passage of the law, shall enter the profession unless he is in possession of a diploma from a reputable dental college. It has long been an idea of mine that when we send out our graduates from the colleges, we should send them out, not only able to perform the necessary operations in their profession, but so well educated in their previous life, as well as in the college, that they would reflect credit upon their alma mater, and do honor to the profession they have chosen. In order to do that it is necessary that all persons who enter a dental college should have a good, general education as a foundation on which to build their professional knowledge in the college and on which to build their reputation in after life. I have, together with a number of my friends in our section of the country, advocated this for a long time, and that there should be a preliminary examination of every person who comes to matriculate in a dental college. But in the condition of affairs in our country, with reference to dental schools, it has been impossible to carry out that idea without making too great a sacrifice, because if a dental school were to set the noble example of requiring a strict preliminary examination, it would cut itself off from a large number of students and the income derived from them, and that is a powerful motive in human nature. However, I think the time is coming, and will soon arrive, when the system of education in our dental schools will be uniform in every respect, uniform in matriculation, length of time in attendance, and in the standard of requirements. In connection with this question there is to be a meeting of the faculties of dental colleges in New York on the 4th of August, and of the National Board of Dental Examiners at Saratoga, on the 8th of August, and I look to see them act, both separately and in concert, in such a manner as to bring about a satisfactory adjustment of these disputed questions, and in such a manner as will inure to the interests of the profession in the direction of the higher dental attainments of which Dr. Palmer speaks.

*Dr. W. H. Atkinson*—I think it is very manifest that we need a higher education of dentists, but the continued reiteration of dental education has become tiresome to me. It is "dental education,"

"dental knowledge," dental this and dental that all the way through, when we don't mean anything of the sort. We mean the education of dentists. If a man has a good English education it is not useful to be continually repeating these phrases, and neglecting the weightier matters of the law, whereby we shall be able to understand the organs upon which we are mainly called to do our work, and thereby bring sorrow or joy to those who are in our hands. You know the enthusiastic hold I have taken upon one person here, (Dr. Heitzman) in his efforts to bring our knowledge to the foundation of our work through histology. Without histology we will never be thoroughly competent dentists. We may have a mass conception, but we will never have a clear discrimination between a healthful and a diseased manifestation of the functions pertaining to the teeth and their connections; if we are to get a higher education, let us take a new deal and ignore the old text-books.

*Dr. M. W. Foster*—This subject of dental education is one upon which I hold peculiar views, that may not coincide with those of some of the gentlemen present, but I am here for the purpose of stating those views and giving other gentlemen an opportunity to combat them. I did not have the pleasure of listening to the gentleman's paper, so that nothing I may say will be personal, or have reference to anything contained in the paper. It seems the height of folly and the merest nonsense to place such eminent gentlemen as I see before me, who have not had the opportunity of getting into a college in their early life, and whose full practice in their later professional life gave them no leisure for it, on the same footing as the boys who come in without the slightest knowledge of dentistry, and require them to study the same length of time before they are permitted to pass the examination. Does it not strike you as a peculiar thing that these eminent practitioners, men who are recognized as our professional brethren, should go to a dental college and stay two years' time, and for what? Is two years sufficient without an examination? Nobody makes that claim. Then what is it after all but the ability to pass a thorough examination that obtains the degree at last. The only question then seems to be as to the abuse of their trust on the part of college faculties, and the means of correcting such abuse is in the hands of your dental societies. Appoint a board of visitors of three or more, whose duty it shall be to attend the examinations of the colleges, and if those gentlemen feel that the examination does not come up to the proper standard, and that the men are not sufficiently advanced to graduate, let them so state and enter their protest; and let the colleges that will not receive and listen to your advisory committees bear the odium of graduating persons who are not fit to practice dentistry.

*Dr. James G. Palmer*—I did not refer to dental education in the colleges in any way; It was not my purpose to do that. I believe that a proper education of young men should be required before they are allowed to enter our offices, and before they are allowed to take up the study of dentistry in any way. That is going down pretty near to the bottom of things, though perhaps not as far back as Dr. Atkinson might like to go. If a man has a thorough classical education he is far better prepared to follow any profession than he is if he lacks that. The man who passes four years in a classical college learns other important things that are not laid down in the curriculum; he becomes imbued with a sort of brotherly feeling and love of his alma mater, and a sense of dignity that a man who spends only a one or two winters' course at a dental school can never have.

*Dr. C. S. Stockton*—The question how to start young men properly for dental work is a great problem. I remember a remark, very wisely made, by Dr. Buckingham, I think, that the colleges did very well with the material that was sent to them. I think that is true. If you should attend the opening session of a dental college and see many of the young men who go there, from their appearance you would judge that it was a pretty hard task to make much out of them in the way of dentistry. I have been very much interested in this subject for a few years past, and I know that this society has done very much to educate and elevate the profession. During the past year the society has taken a step in advance, and we are all proud that New Jersey stands to-day among the first in respect to dental laws. It should be remembered that it is *not* the function of a college to recognize merit wherever it meets with it. A college diploma is not a mere merit-mark. It is not proof of the possession of a definite amount of knowledge. It does not even demonstrate that its holder is a reputable practitioner. It is simply the evidence that he has pursued a prescribed course of study; has gone through a duly enjoined curriculum. The idea that college authorities are the arbiters of the professional standing and actual acquirements of all men, and that it is their province to set their seal upon those who have and those who have not gone through their specific course of study, seems to me very absurd.

*Dr. J. Hayhurst*—Believed there should be some change in the system of dental education, which had been going on in the same rut ever since he could remember almost, and he hoped the National Board of Dental Examiners would, at their approaching meeting, devise some new means of educating dentists; he believed that the old fashioned kind of lectures in dental colleges would be done away with; that the standard of requirement would be the same throughout the land, and that there would be no disposition on the part of one college to find fault with another when diplomas were granted.

*Dr. Carl Heitzman*—Expressed the opinion that the most important element in the education of a dentist was a thorough knowledge of the anatomy of the teeth and their surrounding tissues. It should be the aim of the dentist to know what he does, up to the last point, when he works upon these structures, and that can be done only upon the solid foundation which the histology of the teeth gives. To-day we know that there is not a single tissue that enters into the formation of the teeth that is not alive; we know that live tissues react upon injury done to them; therefore, a foreign substance in the form of a filling that is introduced into a tooth will necessarily cause more or less reaction, and a great deal of harm may be done by the introduction of unsuitable materials in an unscientific way. To avoid such harm and to accomplish the beneficial results that the dentist and his patient desire, a knowledge of the minute anatomy and histology of the teeth is absolutely necessary.

A resolution introduced by Dr. Stockton memorializing the Governor and civil authorities of the State of California in the matter of the imprisonment of Dr. Chalfant for the killing of Dr. Josiah Bacon, to the end that he be pardoned for that offence, was unanimously passed.

At the annual election of officers the following were chosen: President, Dr. J. W. Scarborough, of Lambertville; Vice President, Dr. W. Price Richards, of Orange; Secretary and State Prosecutor, Dr. Chas. A. Meeker, of Newark; Treasurer, Dr. Geo. C. Brown, of Elizabeth.

